# Differential effects of decisional and emotional forgiveness on distress and well-being: A three-wave study of Indonesian adults

**DOI:** 10.3389/fpsyg.2022.918045

**Published:** 2022-10-06

**Authors:** Kaye V. Cook, Ni Made Taganing Kurniati, Christiany Suwartono, Nilam Widyarini, Everett L. Worthington Jr., Richard G. Cowden

**Affiliations:** ^1^Department of Psychology, Gordon College, Wenham, MA, United States; ^2^Faculty of Psychology, Gunadarma University, Depok, West Java, Indonesia; ^3^Faculty of Psychology, Atma Jaya Catholic University of Indonesia, Jakarta, Indonesia; ^4^Department of Psychology, Virginia Commonwealth University, Richmond, VA, United States; ^5^Human Flourishing Program, Harvard University, Cambridge, MA, United States

**Keywords:** forgiveness, culture, Indonesia, health, psychological distress, well-being

## Abstract

Research suggests that interpersonal forgiveness is beneficial to individual functioning, but few longitudinal studies have explored the independent contributions of decisional and emotional forgiveness to reducing different forms of distress and improving multidimensional well-being. In this three-wave (T1: December 2020; T2: January 2021; T3: February 2021) prospective study of predominantly young Indonesian adults (*n* = 595), we examined the associations of decisional and emotional forgiveness with three indicators of distress and 10 components of well-being. Applying the outcome-wide analytic template for longitudinal designs, our primary analysis involved estimating two sets of linear regression models (one set for decisional forgiveness and one set for emotional forgiveness) in which the outcomes were regressed on each interpersonal forgiveness process (one outcome at a time). Adjusting for a range of covariates (including prior values of decisional forgiveness, emotional forgiveness, and all 13 outcomes) assessed at T1, decisional forgiveness assessed at T2 was associated with an increase in seven components of well-being (i.e., life satisfaction, physical health, sense of purpose, promote good, delayed gratification, content with relationships, satisfying relationships) approximately 1 month later at T3. In contrast, emotional forgiveness assessed at T2 was associated with an increase in a single component of well-being (i.e., satisfying relationships) assessed at T3. Neither decisional nor emotional forgiveness assessed at T2 showed evidence of associations with any of the subsequent indicators of distress assessed at T3. Our findings suggest that, at least within a principally collectivistic cultural context such as Indonesia, decisional forgiveness in the aftermath of a transgression may have greater short-term benefits for well-being compared to emotional forgiveness. Implications of the findings for research and interventions are discussed.

## Introduction

Forgiveness is an extensively studied concept that has been shown to reduce distress and increase well-being among people who have been transgressed against (Griffin et al., 2020). Interpersonal forgiveness has been described as an intraindividual process of attitudinal change towards a transgressor—involving cognitions, emotions, and behavioral tendencies—along a continuum ranging from malevolence to benevolence (Worthington and Cowden, 2017; Forster et al., 2020). Worthington emphasizes two components of interpersonal forgiveness—decisional forgiveness and emotional forgiveness (Worthington et al., 2007b, 2020). Decisional forgiveness can be defined as “the behavioral intention statement to treat the transgressor as a person of value, to forswear revenge, and to act in ways that forbear expressions of anger about the transgression” (Worthington and Sandage, 2016, p. 22). Emotional forgiveness is the “emotional transformation from negative unforgiving emotions to some improved state” (Worthington and Sandage, 2016, p. 23). Decisional and emotional forgiveness are two aspects of the same experience, neither one of which necessarily precedes the other or is more cognitive than the other (Exline et al., 2003).

As distinct (though related) aspects of the interpersonal forgiveness process, a reasonable assumption is that decisional and emotional forgiveness would generally not have the same effects on distress and well-being. Decisional forgiveness is associated with reduced hostility and rumination, contributing to better psychological and social well-being (Baker et al., 2017; Kurniati et al., 2017). Emotional forgiveness involves replacing negative affect with positive other-oriented emotions, and therefore may be linked to even more positive effects on distress and well-being than decisional forgiveness (Worthington et al., 2007b; Sun et al., 2014; Webb and Toussaint, 2020). Consistent with this theorizing, existing evidence generally suggests that emotional forgiveness tends to have stronger negative associations with indicators of distress (e.g., depression symptoms, stress) and stronger positive associations with different indicators of well-being (e.g., relationship satisfaction, gratitude) compared to decisional forgiveness (e.g., Chi et al., 2019; Cowden et al., 2019a; Wu et al., 2022). However, not all evidence is consistent with this picture, as some studies have reported stronger correlations with some indicators of distress (e.g., depression symptoms; Mróz et al., 2022) and well-being (e.g., perceived posttraumatic growth; Byra et al., 2022) for decisional forgiveness rather than emotional forgiveness. These findings suggest that emotional forgiveness may not always perform a dominant function in lowering distress and improving well-being.

The growing body of literature on decisional and emotional forgiveness has provided useful insight into the potential benefits of each interpersonal forgiveness process for reducing distress and improving well-being. However, there are several important gaps in knowledge that warrant further attention. First, prior research in this area has relied almost exclusively on cross-sectional designs that are unable to establish a temporal sequence of exposure (e.g., decisional forgiveness) and outcome (e.g., depression symptoms). To better understand the direction of associations between both decisional and emotional forgiveness with distress and well-being, longitudinal studies are needed. Second, previous studies on interpersonal forgiveness, distress, and well-being have largely been conducted with samples from Western cultures that are principally individualistic (Ho, 2020; Sandage et al., 2020). Whereas individualistic cultures tend to emphasize individual autonomy and restoration of intrapersonal equanimity following interpersonal transgressions, collectivistic cultures are known to prioritize relational repair, social harmony, and reconciliation (Hook et al., 2009; Cowden et al., 2019b). This tendency to prioritize the needs of the group (e.g., maintaining social harmony) has been found to be associated with decisional forgiveness in several non-Western contexts that are principally collectivistic, including Nepal and Indonesia (Watkins et al., 2011; Kurniati et al., 2017). Such cultural distinctions may not only shape experiences of interpersonal forgiveness but also associations of decisional and emotional forgiveness with indicators of distress and well-being. Hence, further research is needed on the decisional and emotional processes of interpersonal forgiveness in cultures that are principally collectivistic in orientation (Sandage et al., 2020). Third, previous studies on interpersonal forgiveness (including those involving decisional and emotional forgiveness) have typically focused on one or a few indicators of distress and well-being, thereby providing an incomplete picture of how forgiveness might be related to human functioning (Chen et al., 2019). If we are to develop a more holistic and integrative understanding of the potential benefits of forgiveness, research ought to include a wide range of relevant indicators of distress and well-being simultaneously.

### The present study

To address some of the current gaps in knowledge, we use longitudinal data from a sample of Indonesian adults to examine associations of decisional and emotional forgiveness with 13 indicators of distress and well-being. Applying a rigorous analytic template for longitudinal designs (VanderWeele et al., 2020), we performed two sets of analyses (one for each forgiveness process) to estimate potential causal effects of decisional and emotional forgiveness on each of the outcomes assessed approximately 1 month later. We expected that decisional and emotional forgiveness would generally be associated with lower subsequent distress and higher subsequent well-being, although we anticipated some variation in the strength of associations for each forgiveness process.

Given that a bidirectional link between interpersonal forgiveness and well-being has been proposed but rarely documented (Davis J. L. et al., 2015; Toussaint et al., 2015), we used a similar analytic approach to perform two sets of secondary analyses (one for each forgiveness process) that examined the indicators of distress and well-being as candidate antecedents of subsequent decisional and emotional forgiveness. If one or more candidate antecedents are found to be associated with subsequent decisional or emotional forgiveness, such evidence could be used to develop or refine interventions designed to promote interpersonal forgiveness.

## Materials and methods

### Participants

Data for this study were taken from a three-wave research project focused on self and other-oriented aspects of forgiveness, religion/spirituality, and well-being among Christian and Muslim adults in Indonesia. Using a convenience sampling approach, twelve graduate-level research assistants from four universities located in two major cities in Indonesia recruited participants from their universities (*n* = 311) and local communities (*n* = 309). Interested individuals were provided information about the research and the nature of their prospective involvement, after which they were directed to an online data collection platform where they gave electronic informed consent and then completed the baseline survey (T_1_: 7 to 17 December, 2020). At T_1_, participants responded to a set of sociodemographic items, recalled and briefly wrote about an interpersonal transgression in which they were hurt by another person, and completed a range of well-validated measures. Approximately 1 month and 2 months later, participants who completed the T_1_ survey were invited to respond to the T_2_ (4 to 15 January, 2021) and T_3_ surveys (1 to 13 February, 2021). The T_2_ and T_3_ surveys contained the same set of measures that were administered at T_1_. A masked translation and back-translation process was used to translate the surveys from English to Indonesian (for further details, see Ho et al., 2022); all participants completed the surveys in the Indonesian language. Participants were compensated the equivalent of $6 (USD) for participating in the three surveys.

A total of *N* = 620 participants completed the T_1_ survey. Of those, *n* = 25 (4.03%) were lost to follow-up. Independent samples *t*-tests, Chi-square tests of independence, and Fisher’s exact tests were used to explore differences between the retained participants and those who dropped out at T_1_ (Supplementary Table S1). Participants who dropped out after T_1_ scored lower on religious commitment (*p* = 0.019) and intrinsic religiousness (*p* = 0.021), and they were less likely to report attempted amends-making by transgressors (*p* = 0.032). There was little evidence that the two groups differed on any other covariates, the exposures, or the outcomes (*p*s > 0.05).

Baseline sociodemographic characteristics of participants in the analytic sample (*n* = 595) can be found in Supplementary Table S2. Participants were mostly young adults (*M*_age_ = 21.95, *SD* = 4.39), a majority of whom were female (54.62%) and Javanese or Tionghoa in ethnicity (53.78%). Most of the participants were unmarried (95.63%) and had fulfilled high school equivalency requirements or higher (99.66%). Approximately half of the participants identified as Christian (50.08%), with the remainder identifying as Muslim (49.92%).

## Measures

### Exposures

#### Decisional forgiveness

The six-item Decision to Forgive Scale (DTFS; Davis D. E. et al., 2015) was used to measure the extent to which participants had made a decision to forgive the person who had transgressed against them. Items (e.g., “I have decided to forgive him or her”) are rated using a five-point response scale ranging from 1 (*Strongly disagree*) to 5 (*Strongly agree*). Responses to each item were summed for a total score.

#### Emotional forgiveness

The eight-item Emotional Forgiveness Scale (EFS; Worthington et al., 2007a) measures the degree to which a person has replaced negative other-oriented emotions towards someone who has transgressed against them with emotions that are more positive. Participants responded to the items (e.g., “I care about him or her”) using a five-point response scale ranging from 1 (*Strongly disagree*) to 5 (*Strongly agree*). After reverse scoring three of the items, responses to the items were summed for a total score.

At T_1_, participants responded to the DTFS and EFS after recalling and writing about a specific event in which they were hurt by another person. They were prompted to respond to each measure while considering the interpersonal transgression that they had recently written about. At T_2_ and T_3_, participants were reminded of the interpersonal transgression that they wrote about at T_1_ and asked to respond to the DTFS and EFS while reflecting on that same interpersonal transgression.

#### Outcomes

##### Anxiety symptoms

The Generalized Anxiety Disorder-2 (Kroenke et al., 2007) is a two-item measure of generalized anxiety symptoms. Participants use a four-point response scale (0 = *Not at all*; 3 = *Nearly every day*) to rate each item (e.g., “Feeling nervous, anxious or on edge”). A total score was derived by summing responses to the items.

##### Depression symptoms

The Patient Health Questionnaire-2 (Kroenke et al., 2003) is a two-item measure of depression symptoms. The items (e.g., “Little interest in doing things”) are rated using a four-point response scale (0 = *Not at all*; 3 = *Nearly every day*). Responses to the items were summed for a total score.

##### Suffering

The subjective experience of suffering was measured using the seven-item Personal Suffering Assessment (VanderWeele, 2019). The first item is a global question (i.e., “To what extent are you suffering?”), and the remaining six items ask about different aspects of a person’s experience of suffering (e.g., “The suffering I have been experiencing affects all aspects of my life”). Each item is rated using an 11-point response scale, with different anchor points for the first item (0 = *Not suffering at all*; 10 = *Suffering terribly*) compared to the other six items (0 = *Strongly disagree*; 10 = *Strongly agree*). Consistent with previous research (e.g., Cowden et al., 2021, 2022; Ho et al., 2022), responses to the items were averaged for an overall suffering score.

##### Well-being

The 10-item Flourishing Index (VanderWeele, 2017) assesses near universally valued components of well-being across five domains (i.e., happiness and life satisfaction, mental and physical health, meaning and purpose, character and virtue, close social relationships). The items (e.g., “How satisfied are you with life as a whole these days?”) are rated using an 11-point response scale (from 0 to 10), with orienting labels presented alongside anchor points at each end of the scale (e.g., 0 = *Not satisfied at all*; 10 = *Completely satisfied*). Given that each item is theorized to capture a unique component of well-being, we modeled the 10 items individually.

### Covariates

Based on data that were available, we adjusted for a range of covariates assessed at T_1_. Sociodemographic covariates included age (continuous), gender (female or other, male), ethnic status (Javanese, Tionghoa, other), educational attainment (up to high school equivalency, postsecondary degree or higher), marital status (not married, married), and religious status (Christian, Muslim). We also controlled for transgression-related characteristics known to influence the forgiveness process, including whether the transgressor attempted to make amends (no, yes) and a single-item measure of perceived transgression severity (continuous). Additional covariates (all continuous) included financial and material stability (Secure Flourishing Index; VanderWeele, 2017), trait forgivingness (Trait Forgivingness Scale; Berry et al., 2005), harmonious value (Harmonious Value Scale; Kurniati et al., 2017), religious commitment (Religious Commitment Inventory; Worthington et al., 2003), and intrinsic religiousness (New Indices of Religious Orientation; Francis, 2007). Further details about the measures that were used to assess the covariates can be found in Supplementary Text 1.

### Analytic strategy

#### Preliminary analyses

All statistical processing was performed in R (R Core Team, 2020). Preliminary data screening revealed that fewer than 1% of the retained participants (*n* = 595) had missing data at each timepoint. All subsequent analyses were computed using an available-case approach. We estimated the internal consistency of all multi-item measures using alpha, which we calculated with the *userfriendlyscience* and *psych* packages. Internal consistency values were within acceptable limits (Supplementary Table S2). We computed Pearson correlations with the *apaTables* package to describe the cross-sectional and prospective bivariate associations of decisional and emotional forgiveness with each outcome.

#### Primary analysis

Following the analytic template for outcome-wide longitudinal designs (VanderWeele et al., 2020), we performed two sets of multiple linear regression analyses to model associations of decisional and emotional forgiveness with each of the outcomes (i.e., three indicators of distress and 10 components of well-being). In the first set of models, separate regressions (13 in total) were used to estimate the associations of decisional forgiveness assessed at T_2_ with each T_3_ outcome. The second set of models (13 in total) were identical to the first set, except that we replaced decisional forgiveness assessed at T_2_ with emotional forgiveness assessed at T_2_. Each model adjusted for all T_1_ covariates. All models also controlled for prior values of each outcome variable assessed at T_1_. In addition, we adjusted for both decisional and emotional forgiveness assessed at T_1_. The timepoints from which the covariates, exposures, and outcomes were taken are displayed visually in Supplementary Figure S1. All continuous outcomes were standardized (*M* = 0, *SD* = 1) to facilitate interpretation of the results.

#### Secondary analysis

We used the analytic template for lagged exposure-wide designs (VanderWeele et al., 2020) to perform a secondary analysis in which the three indicators of distress and the 10 components of well-being were each explored as candidate antecedents of decisional and emotional forgiveness. Similar to the primary analysis, we estimated a series of models that involved regressing continuous outcomes of decisional and emotional forgiveness assessed at T_3_ on each candidate antecedent assessed at T_2_ (one candidate antecedent and outcome at a time). All models adjusted for the same T_1_ covariates that were included in the primary analysis, prior values of decisional and emotional forgiveness assessed at T_1_, and prior values of all candidate antecedents assessed at T_1_.

#### Sensitivity analysis

We calculated *E*-values to evaluate the robustness of the results from the primary and secondary analyses to potential unmeasured confounding (VanderWeele and Ding, 2017). *E*-values estimate the minimum strength of association that an unmeasured confounder would need to have with both the exposure and the outcome (on the risk ratio scale), above and beyond the measured covariates, to fully explain away the observed association between the exposure and the outcome. *E*-values can range from 1 to any number greater than 1; higher values indicate that stronger unmeasured confounder risk ratio associations would be needed to explain away the observed exposure-outcome association.

## Results

### Preliminary analyses

Decisional and emotional forgiveness were positively correlated with one another at T_1_, *r* = 0.62, 95% CI [0.56, 0.66], *p* < 0.001. Both were negatively correlated with the indicators of distress and positively correlated with the components of well-being at T_1_ (Supplementary Table S3). Generally, correlations were larger for decisional forgiveness (|*r|* = 0.16 to 0.30) than for emotional forgiveness (|*r|* = 0.09 to 0.22). The correlations of decisional and emotional forgiveness at T_2_ with the outcomes at T_3_ were somewhat smaller than the T_1_ cross-sectional correlations (decisional forgiveness: |*r|* = 0.12 to 0.25; emotional forgiveness: |*r|* = 0.07 to 0.16). The correlations for decisional forgiveness were generally larger than for emotional forgiveness (Supplementary Table S3).

### Primary analysis

Results for the associations of decisional and emotional forgiveness with each subsequent outcome are reported in Table 1. Decisional forgiveness evidenced robust positive associations with three components of well-being, namely sense of purpose (*β* = 0.17, *p* < 0.001), content with relationships (*β* = 0.21, *p* < 0.001), and satisfying relationships (*β* = 0.14, *p* = 0.002). More modest positive associations emerged for four other components of well-being, including life satisfaction (*β* = 0.11, *p* = 0.017), physical health (*β* = 0.11, *p* = 0.031), orientation to promote good (*β* = 0.12, *p* = 0.014), and delayed gratification (*β* = 0.14, *p* = 0.005). There was little evidence to suggest that decisional forgiveness was associated with the three indicators of distress (*β*s = −0.02 to 0.04, *p*s ≥ 0.401) or the other three components of well-being (*β*s = 0.02 to 0.09, *p*s ≥ 0.064).

**Table 1:** Associations of decisional and emotional forgiveness (T_2_) with distress and well-being outcomes assessed 1 month later (T_3_).

Outcome	Exposure			
	Decisional forgiveness		Emotional forgiveness	
	*β* (95% CI)	*E*-values^a^ (EE^b^, LCI^c^)	*β* (95% CI)	*E*-values^a^ (EE^b^, LCI^c^)
*Distress*				
Anxiety symptoms	0.04 (−0.06, 0.14)	(1.24, 1.00)	−0.08 (−0.18, 0.03)	(1.35, 1.00)
Depression symptoms	0.04 (−0.06, 0.14)	(1.24, 1.00)	−0.04 (−0.14, 0.07)	(1.22, 1.00)
Suffering	−0.02 (−0.11, 0.07)	(1.16, 1.00)	−0.04 (−0.13, 0.06)	(1.22, 1.00)
*Well-being*				
Life satisfaction	0.11 (0.02, 0.21)^*^	(1.46, 1.16)	−0.01 (−0.10, 0.09)	(1.08, 1.00)
Happiness	0.09 (−0.01, 0.18)	(1.38, 1.00)	0.06 (−0.04, 0.16)	(1.30, 1.00)
Physical health	0.11 (0.01, 0.21)^*^	(1.44, 1.11)	0.00 (−0.10, 0.11)	(1.05, 1.00)
Mental health	0.07 (−0.02, 0.16)	(1.33, 1.00)	0.02 (−0.07, 0.11)	(1.17, 1.00)
Meaning in life	0.02 (−0.07, 0.10)	(1.13, 1.00)	−0.01 (−0.10, 0.08)	(1.12, 1.00)
Sense of purpose	0.17 (0.08, 0.26)^***^	(1.61, 1.37)	0.04 (−0.06, 0.13)	(1.23, 1.00)
Promote good	0.12 (0.03, 0.22)^*^	(1.49, 1.18)	−0.03 (−0.14, 0.07)	(1.21, 1.00)
Delayed gratification	0.14 (0.04, 0.24)^*^	(1.53, 1.24)	−0.01 (−0.11, 0.09)	(1.11, 1.00)
Content with relationships	0.21 (0.12, 0.30)^***^	(1.71, 1.47)	0.05 (−0.05, 0.14)	(1.26, 1.00)
Satisfying relationships	0.14 (0.05, 0.23)^***^	(1.52, 1.27)	0.09 (0.00, 0.19)^*^	(1.40, 1.05)

*β* = standardized effect size, CI = confidence interval, EE = *E*-value for the effect estimate, LCI = *E*-value for the limit of the confidence interval. *n* = 593 for analyses. In separate models, ordinary least squares regressions were used to regress each outcome on either decisional or emotional forgiveness. Regression models estimate the mean change (*β*) in the standardized scores of each outcome with the change in the exposure. Exposure and outcome variables were continuous and standardized (*M* = 0, *SD* = 1) to facilitate comparison of effect estimates across outcomes. All models adjusted for prior values of age, gender, ethnic status, religious status, marital status, educational attainment, financial and material stability, whether the transgressor attempted to make amends, and perceived transgression severity assessed at T_1_, prior values of both exposure variables (i.e., decisional and emotional forgiveness) assessed at T_1_, and prior values of all outcomes (i.e., all three indicators of distress and all 10 components of well-being) assessed at T_1_. ^*^p < 0.05 before Bonferroni correction, ^***^p < 0.05 after Bonferroni correction (the *p*-value cutoff for Bonferroni correction was 0.05/13 = 0.0038 for each outcome). ^a^The formula for calculating *E*-values can be found in VanderWeele and Ding (2017). ^b^*E*-values for effect estimates are the minimum strength of association that an unmeasured confounder would need to have with both the exposure and the outcome variable to fully explain away the observed effect, after accounting for the measured covariates. ^c^*E*-values for the limit of the 95% CI closest to the null denote the minimum strength of association that an unmeasured confounder would need to have with both the exposure and the outcome variable to shift the confidence interval to include the null value, after accounting for the measured covariates.

We found a modest positive association between emotional forgiveness and a single subsequent component of well-being, namely satisfying relationships (*β* = 0.09, *p* = 0.044). There was little evidence of association between emotional forgiveness and the three indicators of distress (*β*s = −0.08 to −0.04, *p*s ≥ 0.154) or the other nine components of well-being (*β*s = −0.01 to 0.06, *p*s ≥ 0.223).

### Secondary analysis

Results of the secondary analysis involving potential antecedents of subsequent decisional and emotional forgiveness are reported in Table 2. Physical health (*β* = 0.11, *p* = 0.003) and orientation to promote good (*β* = 0.11, *p* = 0.001) evidenced robust positive associations with subsequent decisional forgiveness. Three other components of well-being, namely happiness (*β* = 0.11, *p* = 0.009), mental health (*β* = 0.12, *p* = 0.005), and meaning in life (*β* = 0.10, *p* = 0.020), yielded more modest positive associations with subsequent decisional forgiveness. Depression symptoms (*β* = −0.10, *p* = 0.007) and suffering (*β* = −0.11, *p* = 0.013) yielded modest negative associations with subsequent decisional forgiveness. Anxiety symptoms (*β* = −0.01, *p* = 0.749) and the other five components of well-being (*β*s = 0.05 to 0.08, *p*s ≥ 0.054) showed little evidence of association with subsequent decisional forgiveness.

**Table 2:** Associations of candidate antecedent (*T*_2_) with decisional and emotional forgiveness assessed 1 month later (*T*_3_).

Candidate antecedent	Outcome			
	Decisional forgiveness		Emotional forgiveness	
	*β* (95% CI)	*E*-values (EE, LCI)	*β* (95% CI)	*E*-values (EE, LCI)
*Distress*				
Anxiety symptoms	−0.01 (−0.08, 0.06)	(1.12, 1.00)	−0.05 (−0.12, 0.02)	(1.27, 1.00)
Depression symptoms	−0.10 (−0.17, −0.03)^*^	(1.42, 1.19)	−0.03 (−0.10, 0.04)	(1.18, 1.00)
Suffering	−0.11 (−0.19, −0.02)^*^	(1.44, 1.17)	−0.09 (−0.17, −0.01)^*^	(1.40, 1.12)
*Well-being*				
Life satisfaction	0.05 (−0.03, 0.12)	(1.25, 1.00)	0.07 (−0.00, 0.14)	(1.33, 1.00)
Happiness	0.11 (0.03, 0.18)^*^	(1.43, 1.18)	0.05 (−0.02, 0.13)	(1.28, 1.00)
Physical health	0.11 (0.04, 0.18)^***^	(1.45, 1.22)	0.04 (−0.03, 0.11)	(1.23, 1.00)
Mental health	0.12 (0.04, 0.20)^*^	(1.47, 1.22)	0.05 (−0.03, 0.13)	(1.27, 1.00)
Meaning in life	0.10 (0.02, 0.18)^*^	(1.41, 1.13)	0.09 (0.01, 0.17)*	(1.38, 1.11)
Sense of purpose	0.08 (−0.00, 0.16)	(1.36, 1.00)	0.03 (−0.05, 0.11)	(1.20, 1.00)
Promote good	0.11 (0.05, 0.18)^***^	(1.46, 1.25)	−0.01 (−0.08, 0.05)	(1.12, 1.00)
Delayed gratification	0.07 (−0.00, 0.14)	(1.32, 1.00)	0.07 (−0.00, 0.13)	(1.32, 1.00)
Content with relationships	0.06 (−0.02, 0.13)	(1.30, 1.00)	0.05 (−0.02, 0.12)	(1.28, 1.00)
Satisfying relationships	0.05 (−0.02, 0.13)	(1.28, 1.00)	0.04 (−0.03, 0.12)	(1.24, 1.00)

*β* = standardized effect size, CI = confidence interval, EE = *E*-value for the effect estimate, LCI = *E*-value for the limit of the confidence interval. *n* = 592 for analyses. In separate models, ordinary least squares regressions were used to regress decisional forgiveness or emotional forgiveness on each of the candidate antecedents. Regression models estimate the mean change (*β*) in the standardized scores of each outcome with the change in the candidate antecedent. Candidate antecedents and outcome variables were continuous and standardized (*M* = 0, *SD* = 1) to facilitate comparison of effect estimates across outcomes. All models adjusted for prior values of age, gender, ethnic status, religious status, marital status, educational attainment, financial and material stability, whether the transgressor attempted to make amends, and perceived transgression severity assessed at T_1_, prior values of each candidate antecedent (i.e., all three indicators of distress and all 10 components of well-being) assessed at T_1_, and prior values of the outcomes (i.e., decisional and emotional forgiveness) assessed at T_1_. ^*^p < 0.05 before Bonferroni correction, ^***^p < 0.05 after Bonferroni correction (the *p*-value cutoff for Bonferroni correction was 0.05/13 = 0.0038 for each outcome).

Suffering evidenced a modest negative association with subsequent emotional forgiveness (*β* = −0.09, *p* = 0.025), whereas a modest positive association was found between meaning in life and subsequent emotional forgiveness (*β* = 0.09, *p* = 0.027). We found little evidence to suggest that the other two indicators of distress (*β*s = −0.05 to −0.03, *p*s ≥ 0.145) and other nine components of well-being (*β*s = −0.01 to 0.07, *p*s ≥ 0.056) were associated with subsequent emotional forgiveness.

### Sensitivity analysis

*E*-values for the primary and secondary analyses are reported in Tables 1 and 2, respectively. *E*-values suggested that some of the observed associations were at least modestly robust to potential unmeasured confounding. For example, in the primary analysis, *E*-values for the effect estimates ranged from 1.13 to 1.71 for decisional forgiveness and from 1.05 to 1.40 for emotional forgiveness. *E*-values for the limit of the confidence intervals from the primary analysis were lower, but some associations appeared to be somewhat robust to residual confounding. A similar pattern emerged for the *E*-values corresponding with the secondary analysis.

## Discussion

Previous research on decisional and emotional processes of interpersonal forgiveness, distress, and well-being has typically relied on cross-sectional data from samples living in contexts characterized principally as individualistic in cultural orientation. In the present study, we sought to extend this body of empirical literature by estimating the short-term effects of decisional and emotional forgiveness on 13 indicators of distress and well-being in a sample of Indonesian adults. The findings were partially consistent with our expectations, in that both decisional and emotional forgiveness were associated with improved subsequent well-being on one or more components that were assessed. Decisional forgiveness evidenced stronger and more consistent associations with subsequent components of well-being than emotional forgiveness, but neither forgiveness process showed evidence of associations with any of the indicators of distress. These findings highlight the utility of examining decisional and emotional forgiveness as unique processes in the experience of interpersonal forgiveness, and they demonstrate the potential short-term benefits of these distinct forgiveness processes for various domains of human functioning.

### Decisional forgiveness, emotional forgiveness, and subsequent outcomes

Whereas decisional forgiveness was associated with subsequent improvements in seven of the 10 components of well-being (i.e., life satisfaction, physical health, sense of purpose, promote good, delayed gratification, content with relationships, satisfying relationships), emotional forgiveness was associated with improved subsequent well-being on a single outcome (i.e., satisfying relationships). Our findings generally diverge from earlier studies that have reported evidence of stronger associations with different indicators of well-being (e.g., satisfaction with relationships, character strengths) for emotional forgiveness relative to decisional forgiveness (e.g., Chi et al., 2019; Wu et al., 2022), although some evidence of stronger associations for decisional forgiveness has also been reported for select indicators of well-being (e.g., perceived posttraumatic growth; Byra et al., 2022). There could be several potential explanations for the dissimilarities between the pattern of findings in our study and those reported previously, such as differences in sample characteristics (e.g., participants from cultures that are more vs. less collectivistic), research design (e.g., cross-sectional vs. longitudinal), and analytic decisions (e.g., more vs. less comprehensive adjustment for potential confounders). In contrast with earlier research, the present study is among the first to apply a rigorous analytic approach to estimate the effects of both the decisional and emotional processes of interpersonal forgiveness on a range of distress and well-being outcomes simultaneously.

Previous research has shown that emotional forgiveness seems to be an especially effective strategy for coping with the stress of being transgressed against within cultures that are principally individualistic in orientation (Worthington et al., 2007b), but our findings suggest that decisional forgiveness could be a more effective short-term strategy for supporting individual well-being within collectivistic dominant cultures. In a collectivistic culture such as Indonesia, where maintenance of social harmony and interpersonal reconciliation are prioritized (Joo et al., 2019; Worthington et al., 2020), victims who emphasize processing of decisional forgiveness over emotional forgiveness might experience greater benefits for their well-being in the short-term because their post-transgression response is more congruent with social norms and expectations (Sandage et al., 2020). However, it is important to acknowledge that the pattern of findings observed in this study could be a function of the one-month lag between assessments of the exposures and outcomes, and there is a possibility that the relative effects of decisional and emotional forgiveness on well-being may change over time. For example, decisional forgiveness may have stronger short-term implications for well-being because making a decision to forgive can transpire relatively rapidly, whereas it may take longer for the salutary effects of emotional forgiveness to emerge because this process tends to require more time (Worthington et al., 2007b). This kind of temporal trend may be particularly likely in collectivistic cultures because decisional forgiveness tends to be a higher priority than emotional forgiveness (Watkins et al., 2011; Hook et al., 2013).

Neither decisional nor emotional forgiveness were found to be associated with subsequent anxiety symptoms, depression symptoms, or suffering. These findings largely diverge from prior work that suggests both decisional and emotional forgiveness tend to be related to lower distress (e.g., Kurniati et al., 2017; Cowden et al., 2019a), although mixed evidence has been documented. For example, Mróz et al. (2022) found that certain indicators of distress were uncorrelated with either one (e.g., depression symptoms) or both (e.g., negative affect) interpersonal forgiveness processes. Our findings could be explained by the one-month lag between assessments, as more time might be needed to find evidence of associations between each interpersonal forgiveness process and the indicators of distress that were examined. An alternative but complementary possibility is that decisional and emotional forgiveness may be less closely related to distress in cultures that are more collectivistic. For example, Markus and Kitayama (1991) suggest that people with interdependent selves, thought to be more common among those in collectivistic cultures, may be better at tolerating or inhibiting the experience of negative emotions. Indonesians may be more inclined to avoid the negative emotional responses (e.g., anger, resentment) that typify the post-transgression experiences of people in cultures that are principally individualistic, particularly if the transgression occurs within the context of a close relationship. Cross-cultural longitudinal studies that enable robust comparisons to be made between samples that vary on the individualism–collectivism spectrum could contribute to evaluating this possibility.

### Antecedents of decisional and emotional forgiveness

Our secondary analysis indicated that one or more component of well-being was associated with a higher likelihood of subsequent decisional and emotional forgiveness approximately 1 month later. Similar to the findings of the primary analysis, more components of well-being were associated with decisional forgiveness (i.e., happiness, physical health, mental health, meaning in life, promote good) compared to emotional forgiveness (i.e., meaning in life). We also found that depression symptoms were associated with lower subsequent decisional forgiveness, and suffering was associated with lower subsequent decisional and emotional forgiveness. Our findings suggest that suffering and meaning in life may be two particularly useful targets of interventions to promote interpersonal forgiveness in the short-term, given that each was associated with both subsequent decisional and emotional forgiveness.

Contrasting the findings of the primary and secondary analyses, only two of the 13 candidate antecedents (i.e., physical health, orientation to promote good) showed evidence of a bidirectional association with decisional forgiveness, and we did not find any evidence of bidirectional associations for emotional forgiveness. Although these findings provide some support for theorizing that suggests interpersonal forgiveness and well-being are reciprocally related (Davis J. L. et al., 2015; Toussaint et al., 2015), most of the associations involving the interpersonal forgiveness processes and the outcomes included in this study were unidirectional. Taken together, this study’s findings suggest that people who experience a reduction in some forms of distress and increases in some components of well-being may be more likely to process interpersonal forgiveness, which could precipitate further improvements in well-being.

### Implications for research and interventions

The common narrative is that interpersonal forgiveness leads a person to be more well (Toussaint et al., 2020; Webb and Toussaint, 2020). Our findings have shown that the positive implications of forgiving others may be far more expansive than merely leading a person to be more well, including a greater sense of purpose in life, growth in character and virtue, and better social relationships. Therefore, evidence-based interventions designed to promote interpersonal forgiveness could produce a diffuse range of salubrious consequences. Previous research on the efficacy of interpersonal forgiveness interventions has largely focused on a narrow range of outcomes beyond forgiveness itself, typically those concerning psychological health (e.g., depression symptoms; for a review, see Wade and Tittler, 2020). The findings of this study suggest it may be useful for intervention research to broaden the scope of outcomes to potentially relevant domains of human life that have been underemphasized or overlooked (e.g., physical health).

Based on the findings of this study, interventions and other resources aimed at the promotion of interpersonal forgiveness among Indonesian adults might be particularly effective in supporting well-being over the short-term if they encourage people to make a decision to forgive under the right circumstances. Such circumstances might not include situations in which the transgressor is likely to take advantage of the forgiver (McCullough, 2008; Cowden et al., 2019a), unless strict controls can be exercised over the transgressor’s subsequent behavior by a third party capable of regulating behavior. Although decisional forgiveness might be the initial priority of culturally sensitive forgiveness interventions for Indonesian adults, more thorough processing of interpersonal forgiveness will require an emphasis on emotional forgiveness as well (Kurniati et al., 2017). One promising culturally sensitive forgiveness intervention that has received some support for use with Indonesians is the REACH Forgiveness curriculum modified for collectivistic contexts (i.e., REACH forgiveness collectivistic; Kurniati et al., 2020), which attempts to strike a culturally appropriate balance between increasing the likelihood of decisional forgiveness whilst guiding individuals through processing of emotional forgiveness. Further testing and possible refinement of the REACH forgiveness collectivistic intervention is likely to augment preliminary research that has documented evidence of its utility in promoting interpersonal forgiveness among Indonesians.

### Limitations and future research directions

The present study has several methodological limitations. Our findings are based on data from a nonrepresentative sample of Indonesian adults. Although this study represents an important step in enriching our understanding of interpersonal forgiveness and its implications for human functioning in non-Western contexts, additional evidence is required to determine whether our findings are generalizable to the wider population of adults in Indonesia. Further research is also needed to assess the transportability of the findings to other collectivistic contexts, including those that are more versus less comparable to the sociocultural composition of the Indonesian population. A key strength of this study is that we examined several distress and well-being outcomes using measures that are well-validated and appropriate for use in a wide range of populations, but one of the drawbacks of our broad assessment approach is that the outcomes were measured using one or two items each. Therefore, this study’s findings should be considered in light of the narrow conceptual coverage of the measures that were used to assess the outcomes. In addition, all variables were assessed *via* self-report, which may be subject to bias (e.g., socially desirable responding). Our longitudinal design included a one-month lag between assessments, and the findings provide a snapshot of associations between decisional and emotional processes of interpersonal forgiveness, indicators of distress, and components of well-being at a single point in time. A longer interval between assessments may be needed to observe effects of both decisional and emotional forgiveness on many of the outcomes that were of interest in this study. Moreover, patterns of associations between these two interpersonal forgiveness processes and the outcomes may change over time. Future cohort studies could build on the findings of this study by including additional follow-ups and longer lags between assessments. In contrast with most prior observational studies on decisional and emotional processes of interpersonal forgiveness, we used three waves of data that enabled us to establish a temporal order between variables. We also attempted to reduce concerns about unmeasured confounding and reverse causation by adjusting for relevant covariates that were available in the dataset, including prior values of both the exposure and outcome variables, and *E*-values suggested that many of the results were at least modestly robust to residual confounding. However, we cannot completely rule out the possibility that the results of this study might be confounded by unmeasured factors (e.g., personality traits).

## Conclusion

Existing empirical research on interpersonal forgiveness suggests that emotional forgiveness tends to be more closely associated with better individual well-being than decisional forgiveness. In this prospective study of Indonesian adults, we found evidence indicating that decisional forgiveness in the aftermath of a transgression may have greater short-term benefits for well-being compared to emotional forgiveness. Although this imbalance in the short-term effects of decisional and emotional forgiveness on well-being does not imply that emotional forgiveness is less relevant or necessary among Indonesians, it does suggest that there may be circumstances in which it could be especially useful to encourage Indonesians (and perhaps other individuals living in comparable sociocultural contexts) to make a decision to forgive a transgressor. As the benefits of granting decisional forgiveness for well-being accrue in the short-term, that might set the stage for more and faster emotional forgiveness to unfold, which could have other favorable consequences for well-being that emerge over a longer period of time.

## Data availability statement

All data related to this study are publicly available on the Open Science Framework (https://osf.io/xwvur/).

## Ethics statement

This study was reviewed and approved by Nusantara Scientific Psychology Consortium (015/2020 Etik/KPIN). Individuals were provided information about the research and the nature of their prospective involvement, then directed to an online data collection platform where they provided electronic informed consent.

## Author contributions

KC, TK, CS, and NW were responsible for conceptualization, data curation, funding acquisition, investigation, methodology, and project administration. KC was responsible for writing the initial draft of the manuscript. EW and RC provided analytic support and assisted with editing the manuscript. All authors contributed to the article and approved the submitted version.

## Funding

This work was supported by a Global Religion Research Initiative grant from the Templeton Religion Trust to the Global Religious Research Initiative, PTE Prime Award no. TRT0118, Subaward no. 262164.

## Conflict of interest

The authors declare that the research was conducted in the absence of any commercial or financial relationships that could be construed as a potential conflict of interest.

## Publisher’s note

All claims expressed in this article are solely those of the authors and do not necessarily represent those of their affiliated organizations, or those of the publisher, the editors and the reviewers. Any product that may be evaluated in this article, or claim that may be made by its manufacturer, is not guaranteed or endorsed by the publisher.

## Supplementary material

The Supplementary material for this article can be found online at: https://www.frontiersin.org/articles/10.3389/fpsyg.2022.918045/full#supplementary-material
