# Supplementary material for: Differential effects of decisional and emotional forgiveness on distress and well-being: A three-wave study of Indonesian adults
Source: Front Psychol. 2022 Oct 6;13:918045. doi: 10.3389/fpsyg.2022.918045 (PMC9582772; doi:10.3389/fpsyg.2022.918045)
Supplement: Supplementary file 1 [file Presentation_1.pdf]

Differential Effects of Decisional and Emotional Forgiveness on Distress and Well-being: A Three-Wave Study of Indonesian Adults

SUPPLEMENTARY MATERIAL

Supplemental Text 1: Covariate Assessment

Transgressor Made Amends

At T_1, participants used a dichotomous response format (0 = *No*; 1 = *Yes*) to indicate whether the transgressor had attempted to make amends for the hurt that they recalled and briefly wrote about (i.e., "Did the person attempt to make amends [e.g., by offering an apology]?").

Perceived Transgression Severity

At T_1, participants were asked to rate the severity of the transgression that they recalled and briefly wrote about (i.e., "Please rate the severity of the offense") using a five-point response format (1 = *Not at all severe*; 5 = *Very severe*).

Financial and Material Stability

Participants completed both items (i.e., "How often do you worry about being able to meet normal monthly living expenses?" and "How often do you worry about safety, food, or housing?") from the Financial and Material Stability domain of the Secure Flourishing Index (VanderWeele, 2017), each of which was rated using an 11-point response format (0 = *Worry all of the time*; 10 = *Do not ever worry*). The items were averaged for a total score.

Trait Forgivingness

The 10-item Trait Forgivingness Scale (Berry et al., 2005) was used to assess an individual's tendency to forgive others across time and situations (e.g., "I am a forgiving person"). Items are rated using a five-point response format (1 = *Strongly disagree*; 5 = *Strongly agree*). After reverse scoring relevant items, responses are summed for a total score.

Harmonious Value

We used the nine-item Harmonious Value Scale (Kurniati et al., 2017) to assess a person's tendency to value maintaining social harmony and avoiding rumination. The items (e.g., "It's important to resolve any interpersonal conflicts immediately") are rated on a five-

point response scale ranging from 1 (*Strongly disagree)* to 5 (*Strongly agree)*. Responses to the items are summed for a total score.

Religious Commitment

Participants responded to the Religious Commitment Inventory-10 (Worthington et al., 2003). The 10-item measure assesses the extent to which an individual is involved in his/her religion (e.g., "I often read books and magazines about my faith"), and it has been validated for use with different religious traditions (including Christianity and Islam). Items are rated using a five-point response scale ranging from 1 (*Not at all true of me*) to 5 (*Totally true of me*). Responses to the items are summed for a total score.

Intrinsic Religiousness

We used the six-item Intrinsic subscale of the New Indices of Religious Orientation (Francis, 2007). The measure assesses the extent to which individuals integrate their religious faith into all parts of their lives, value personal religious practices to connect with God, and prioritize public religious practices to signal their commitment to God (e.g., "My religious beliefs really shape my whole approach to life"). A five-point response scale is used to rate each item (1 = *Disagree strongly*; 5 = *Agree strongly*). Responses to the items are summed for a total score.

Table S1

Baseline Characteristics of Participants Who Remained in the Cohort and Participants Who Were Lost to Follow-up

Characteristic	Retained (n = 595)		Lost to follow-up (n = 25)		p-value
	n (%)	$M \pm SD$	n (%)	$M \pm SD$	
**Covariates**					
Age		21.95 ± 4.39		21.92 ± 2.41	.976
Gender					
Female or other	326 (54.79)		10 (40.00)		.212
Male	269 (45.21)		15 (60.00)		
Ethnic status					
Javanese	170 (28.57)		6 (24.00)		.628
Tionghoa	150 (25.21)		5 (20.00)		
Other	275 (46.22)		14 (56.00)		
Educational attainment					1.000
Up to high school equivalency	401 (67.39)		17 (68.00)		
Postsecondary degree or higher	194 (32.61)		8 (32.00)		
Marital status					1.000
Not married	569 (95.63)		24 (96.00)		
Married	26 (4.37)		1 (4.00)		
Religious status					.695
Christian	298 (50.08)		11 (44.00)		
Muslim	297 (49.92)		14 (56.00)		
Transgressor made amends					.032
No	381 (64.03)		21 (87.50)		
Yes	214 (35.97)		3 (12.50)		
Perceived transgression severity		3.97 ± 1.03		4.33 ± 1.05	.094
Financial and material stability		5.61 ± 2.55		5.32 ± 2.36	.579
Trait forgivingness		33.98 ± 6.15		31.67 ± 6.85	.073
Harmonious value		44.08 ± 4.97		42.67 ± 6.29	.176
Religious commitment		31.40 ± 8.79		27.16 ± 9.60	.019
Intrinsic religiousness		24.15 ± 4.13		22.17 ± 3.87	.021
**Exposures**					
Decisional forgiveness		23.57 ± 5.07		22.21 ± 5.41	.199
Emotional forgiveness		22.80 ± 5.61		21.92 ± 4.60	.446
**Outcomes**					
Distress					
Anxiety symptoms		2.41 ± 1.66		2.24 ± 1.76	.609
Depression symptoms		2.36 ± 1.66		2.20 ± 1.15	.631
Suffering		3.69 ± 2.41		3.89 ± 2.02	.676
Well-being					
Life satisfaction		6.21 ± 2.17		5.92 ± 2.38	.521
Happiness		6.48 ± 2.02		6.48 ± 2.40	.998
Mental health		6.27 ± 2.28		6.64 ± 2.36	.428
Physical health		7.06 ± 1.88		7.36 ± 2.12	.438
Meaning in life		6.63 ± 2.26		6.08 ± 2.27	.233
Sense of purpose		6.81 ± 2.37		6.52 ± 2.73	.554
Promote good		8.14 ± 1.62		8.32 ± 1.55	.588
Delayed gratification		7.54 ± 1.94		7.64 ± 2.06	.807
Content with relationships		7.37 ± 2.04		7.48 ± 1.58	.793
Satisfying relationships		6.74 ± 2.12		6.52 ± 1.98	.615

Note. M = mean, SD = standard deviation. Percentages refer to the proportion of individuals within each inclusion category with that characteristic. p-values come from independent samples t-tests, χ^2, or Fisher's exact tests that were used to examine the mean (SD) levels of the characteristic or the proportion of individuals within each category with that characteristic.

Table S2

Distribution of Participant Characteristics in the Analytic Sample

Variable	*n* (%)	*M* ± *SD*	α [95% CI]
**Time 1**			
Age, years (range: 18–55)		21.95 ± 4.39	
Gender			
Female	325 (54.62)		
Male	269 (45.21)		
Other	1 (0.17)		
Ethnic status			
Javanese	170 (28.57)		
Tionghoa	150 (25.21)		
Other	275 (46.22)		
Educational attainment			
Up to high school equivalency	401 (67.39)		
Postsecondary degree or higher	194 (32.61)		
Marital status			
Not married	569 (95.63)		
Married	26 (4.37)		
Religious status			
Christian	298 (50.08)		
Muslim	297 (49.92)		
Transgressor made amends			
No	381 (64.03)		
Yes	214 (35.97)		
Perceived transgression severity (range: 1–5)		3.97 ± 1.03	
Financial and material stability (range: 0–10)		5.61 ± 2.55	.75 [.72, .78]
Trait forgivingness (range: 11–50)		33.98 ± 6.15	.81 [.79, .83]
Harmonious value (range: 23–54)		44.08 ± 4.97	.79 [.77, .81]
Religious commitment (range: 10–50)		31.40 ± 8.79	.91 [.90, .92]
Intrinsic religiousness (range: 6–30)		24.15 ± 4.13	.86 [.84, .88]
Decisional forgiveness (range: 6–30)		23.57 ± 5.07	.93 [.92, .94]
Emotional forgiveness (range: 8–40)		22.80 ± 5.61	.79 [.77, .81]
Anxiety symptoms (range: 0–6)		2.41 ± 1.66	.80 [.78, .82]
Depression symptoms (range: 0–6)		2.36 ± 1.66	.64 [.60, .68]
Suffering (range: 0–10)		3.69 ± 2.41	.93 [.92, .94]
Life satisfaction (range: 0–10)		6.21 ± 2.17	
Happiness (range: 0–10)		6.48 ± 2.02	
Mental health (range: 0–10)		6.27 ± 2.28	
Physical health (range: 0–10)		7.06 ± 1.88	
Meaning in life (range: 0–10)		6.63 ± 2.26	
Sense of purpose (range: 0–10)		6.81 ± 2.37	
Promote good (range: 0–10)		8.14 ± 1.62	
Delayed gratification (range: 0–10)		7.54 ± 1.94	
Content with relationships (range: 0–10)		7.37 ± 2.04	
Satisfying relationships (range: 0–10)		6.74 ± 2.12	
**Time 2**			
Decisional forgiveness (range: 6–30)		23.94 ± 4.74	.94 [.93, .95]
Emotional forgiveness (range: 8–40)		23.72 ± 5.42	.81 [.79, .83]
Anxiety symptoms (range: 0–6)		2.20 ± 1.67	.82 [.80, .84]
Depression symptoms (range: 0–6)		2.13 ± 1.62	.72 [.69, .75]
Suffering (range: 0–10)		3.66 ± 2.31	.95 [.94, .96[
Life satisfaction (range: 0–10)		6.47 ± 2.14	
Happiness (range: 0–10)		6.41 ± 2.04	

Mental health (range: 0–10)	6.48 ± 2.03	
Physical health (range: 0–10)	7.06 ± 1.92	
Meaning in life (range: 0–10)	6.71 ± 2.14	
Sense of purpose (range: 0–10)	6.90 ± 2.23	
Promote good (range: 0–10)	7.91 ± 1.62	
Delayed gratification (range: 0–10)	7.34 ± 1.83	
Content with relationships (range: 0–10)	7.34 ± 1.80	
Satisfying relationships (range: 0–10)	6.86 ± 1.95	
**Time 3**		
Decisional forgiveness (range: 6–30)	24.12 ± 5.02	.96 [.96, .96]
Emotional forgiveness (range: 8–40)	24.30 ± 5.54	.80 [.78, .82]
Anxiety symptoms (range: 0–6)	2.12 ± 1.64	.87 [.85, .88]
Depression symptoms (range: 0–6)	2.07 ± 1.69	.80 [.78, .82]
Suffering (range: 0–10)	3.73 ± 2.33	.95 [.94, .96]
Life satisfaction (range: 0–10)	6.64 ± 2.13	
Happiness (range: 0–10)	6.61 ± 2.04	
Mental health (range: 0–10)	6.61 ± 1.99	
Physical health (range: 0–10)	7.11 ± 1.81	
Meaning in life (range: 0–10)	6.76 ± 2.14	
Sense of purpose (range: 0–10)	6.90 ± 2.24	
Promote good (range: 0–10)	7.82 ± 1.64	
Delayed gratification (range: 0–10)	7.33 ± 1.79	
Content with relationships (range: 0–10)	7.34 ± 1.81	
Satisfying relationships (range: 0–10)	6.96 ± 1.91	

Note. CI = confidence interval, *M* = mean, *SD* = standard deviation, α = alpha (estimated internal consistency).

Table S3

Cross-sectional and Prospective Pearson Correlations of Decisional and Emotional Forgiveness with Distress and Well-being Outcomes

Outcome (T_1)	Decisional forgiveness (T_1)	Emotional forgiveness (T_1)
**Distress**		
Anxiety symptoms	-.17 [-.24, -.09]***	-.17 [-.25, -.10]***
Depression symptoms	-.23 [-.30, -.15]***	-.17 [-.25, -.09]***
Suffering	-.22 [-.30, -.15]***	-.16 [-.23, -.08]***
**Well-being**		
Life satisfaction	.26 [.18, .33]***	.21 [.13, .29]***
Happiness	.26 [.19, .34]***	.22 [.14, .29]***
Mental health	.28 [.20, .35]***	.22 [.14, .29]***
Physical health	.18 [.10, .26]***	.15 [.07, .23]***
Meaning in life	.24 [.17, .32]***	.17 [.09, .24]***
Sense of purpose	.19 [.12, .27]***	.09 [.01, .17]*
Promote good	.30 [.22, .37]***	.19 [.11, .26]***
Delayed gratification	.16 [.08, .23]***	.11 [.02, .18]*
Content with relationships	.24 [.16, .31]***	.16 [.08, .24]***
Satisfying relationships	.30 [.23, .37]***	.19 [.12, .27]***
Outcome (T_3)	Decisional forgiveness (T_2)	Emotional forgiveness (T_2)
**Distress**		
Anxiety symptoms	-.12 [-.20, -.04]**	-.16 [-.24, -.08]***
Depression symptoms	-.14 [-.21, -.06]***	-.15 [-.23, -.07]***
Suffering	-.19 [-.26, -.11]***	-.12 [-.20, -.04]**
**Well-being**		
Life satisfaction	.23 [.15, .31]***	.10 [.02, .18]*
Happiness	.22 [.14, .30]***	.12 [.04, .20]**
Mental health	.23 [.16, .31]***	.16 [.08, .23]***
Physical health	.24 [.16, .31]***	.13 [.05, .21]**
Meaning in life	.19 [.12, .27]***	.10 [.02, .18]*
Sense of purpose	.23 [.16, .31]***	.10 [.02, .18]*
Promote good	.22 [.15, .30]***	.07 [-.01, .15]
Delayed gratification	.23 [.15, .30]***	.08 [-.00, .15]
Content with relationships	.25 [.18, .33]***	.13 [.05, .21]**
Satisfying relationships	.21 [.14, .29]***	.15 [.07, .22]***

Note. *$p < .05$, **$p < .01$, ***$p < .001$.

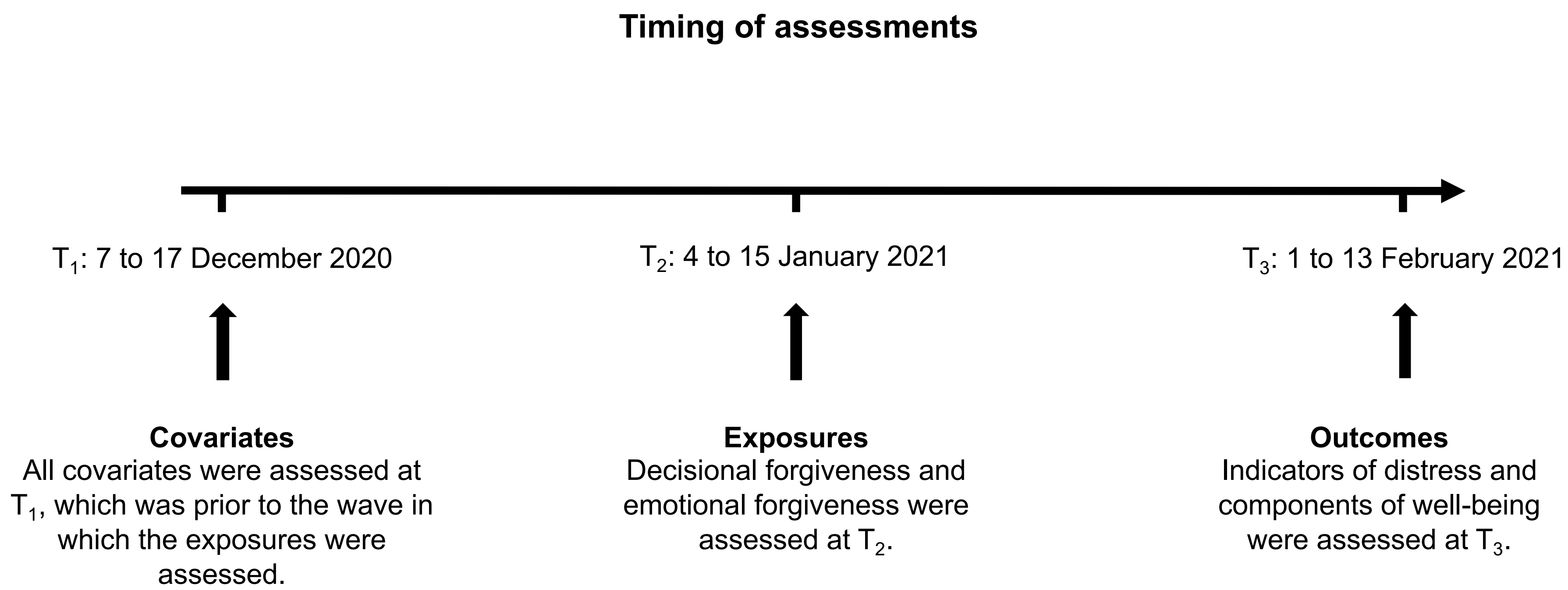

Figure S1. Visual Depiction of Assessment Timings for Covariate, Exposure, and Outcome Variables in the Primary Analysis.

References

Berry, J. W., Worthington, E. L., Jr., O'Connor, L. E., Parrott, L., III, and Wade, N. G. (2005). Forgivingness, vengeful rumination, and affective traits. J. Pers. 73(1), 183–226. doi: 10.1111/j.1467-6494.2004.00308.x

Francis, L. J. (2007). Introducing the New Indices of Religious Orientation (NIRO): conceptualization and measurement. Ment. Health Relig. Cult., 10(6), 585–602. doi: 10.1080/13674670601035510

Kurniati, N. M. T., Worthington, E. L., Jr., Poerwandari, E. K., Ginanjar, A. S., and Dwiwardani, C. (2017). Forgiveness in Javanese collective culture: the relationship between rumination, harmonious value, decisional forgiveness and emotional forgiveness. Asian J. Soc. Psychol. 20(2), 113–127. doi: 10.1111/ajsp.12173

VanderWeele, T. J. (2017). On the promotion of human flourishing. Proc. Natl. Acad. Sci. 114(31), 8148–8156. doi: 10.1073/pnas.1702996114

Worthington, E. L., Jr., Wade, N. G., Hight, T. L., Ripley, J. S., McCullough, M. E., Berry, J. W., et al. (2003). The Religious Commitment Inventory--10: development, refinement, and validation of a brief scale for research and counseling. J. Couns. Psychol. 50(1), 84–96. doi: 10.1037/0022-0167.50.1.84
